# Medical Education in Scotland

**Published:** 1894-09-29

**Authors:** 


### Medical Education in Scotland.

Ask a Scotsman why it is that young men from all parts of England, London included, from India, Australia, America, from every country where English is spoken, resort to Edinburgh, Glasgow, Aberdeen, St. Andrews, and Dundee for their education in medicine, and he will tell you without hesitation that it is because the education provided at these schools is of the very best. That the education is good is no doubt one of the reasons; another, which must be allowed to be powerful, is that it is cheap. Fees are low, the cost of living is less than it is in England. Moreover, Scotland offers University degrees on terms that are not beyond the reach of the youth of average intellect of respectable education. This is an important consideration, for with the British public the title Doctor carries much weight. These things considered, it is not surprising that Scotland finds great favour with those who are about to enter upon the study of medicine, nor is there any reason to regret that it should be so.

Upon one point, however, a wrong impression is very general. Parents who have a lively sense of the dangers that beset the medical student in a great city like London, are apt to think that in a quiet Scottish town their sons will be as much out of harm's way as in a country village. This is a great mistake. The small town has temptations of its own which in some respects are even more insidious than those of the great city. Moreover, it is one of the features of undergraduate life in Scotland, that from the first the student is thrown upon his own resources. When he is not at classes he does what he likes. Many of the schools—those of the Universities of Edinburgh and Glasgow for example—are so large that close personal supervision is really impossible. These things are not stated in order to deter parents from sending their lads to Scotland, but to dispel an illusion which it is within the writer's knowledge has often had serious, sometimes tragic, results. Young men have been ruined for life in distant northern towns while their parents in the south have never suspected that anything was wrong, have never even troubled to inquire whether it was well with their sons.

Of the four centres of medical education in Scotland—for Dundee and St. Andrews may be counted as one—Edinburgh has the greatest reputation, and is the most frequented. Medicine and surgery in Edinburgh have a history of which every Scotsman is proud, and although the days of Syme and Simpson are over, the teachers who are now in office worthily maintain the traditions of the past. The number of medical students at the University of Edinburgh is very large, and were it not that the extra-mural schools relieve the pressure somewhat, it would be difficult to meet the demands upon space and teaching power. The schools of medicine at Surgeons' Hall and Minto House are excellent institutions doing admirable work. To their success a spirit of healthy rivalry with the University has no doubt contributed, while the fact that the lectures at these schools qualify for the University degrees has proved effective in maintaining the standard of University teaching. There is a school of medicine for women in Edinburgh, of which Dr. Sophia Jex-Blake is Dean; and there are special classes for women at Surgeons' Hall. Edinburgh students receive clinical instruction at the Royal Infirmary, one of the finest as, with its 720 beds, it is the second largest, of the hospitals of this country.

A city which has claims to be regarded as the second largest in the kingdom offers, it need hardly be said, abundant material for the study of disease. Glasgow has two great hospitals, the Royal Infirmary, the older of the two, with about 600 beds, and the Western Infirmary with 400. The new University buildings are close to the Western Infirmary, where the University students receive their clinical instruction. Glasgow alone of the Scottish Universities has provided for the medical education of women. At St. Margaret's College there is a full course of classes for women taught by University teachers and qualifying for the University degrees. Glasgow has several flourishing extra-mural schools. Anderson's College is able to boast that it has given twelve professors to the University of Glasgow, of whom four at present hold office. St. Mungo's College is of recent origin. It occupies new buildings erected for the purposes of a medical school adjoining and communicating with the Royal Infirmary, which is Glasgow's largest hospital.

The University of St. Andrews has long had the right to confer degrees in medicine. There was a time when these degrees were of no special value; but it is only right to say that all that is changed now. Some years ago University College, Dundee, was founded, and this thriving institution has been incorporated with the University of St. Andrews. The Dundee Royal Infirmary contains 286 beds, and offers exceptional facilities for practical work. University College, thanks to the public spirit of Dundee, is excellently equipped, and for the first three years of the medical curriculum full provision is made. At St. Andrews several science classes are held. The examiners for the St. Andrew's University degrees in medicine are all men of high repute.

Although Aberdeen is the furthest north of the centres of medical education in Britain, an Aberdeen degree and an Aberdeen training are held in the highest respect all over the English-speaking world. The distinctive mark of the teaching at Aberdeen University is thoroughness rather than brilliancy. Hence it is that Aberdeen men have a character for reliability, which has stood them in excellent stead. The University buildings are at present undergoing extensive alterations, which will contribute greatly not only to the efficiency of the medical school, but to the amenity of the city. The Aberdeen Royal Infirmary has been recently entirely remodelled and substantially enlarged. In addition, the Infirmary, the Royal Lunatic Asylum, with accommodation for over 700 patients, the Aberdeen Dispensary, the Sick Children's Hospital, and the City Fever Hospital afford facilities for clinical study.

